# Calibrated early-warning models with fairness auditing and selective prediction for course withdrawal risk: Evidence from OULAD

**DOI:** 10.1371/journal.pone.0352867

**Published:** 2026-07-15

**Authors:** Suhan Wu, Jingyi Duan, Min Luo

**Affiliations:** 1 School of Economics and Management, Nanjing Polytechnic Institute, Nanjing, China; 2 College of Life and Health, Nanjing Polytechnic Institute, Nanjing, China; 3 School of Management, Shenzhen University of Information Technology, Shenzhen, China; National Chung Hsing University, TAIWAN

## Abstract

Early-warning systems (EWS) in learning analytics are increasingly used to identify learners at risk of course withdrawal, but their deployment-critical properties are often under-reported once predicted scores are converted into intervention policies. This study develops a deployment-oriented evaluation protocol for course-withdrawal risk using the Open University Learning Analytics Dataset (OULAD). An early-window feature set was constructed from the first four weeks of learner activity and evaluated under a group-wise train–test split by course presentation. Multiple classifiers were benchmarked, including logistic regression, histogram-based gradient boosting (HGB), random forest, support vector machine, AdaBoost, K-nearest neighbors, XGBoost, LightGBM, and CatBoost. A calibrated HGB model was then retained as the main probabilistic model for downstream analyses of probability reliability, threshold sensitivity, subgroup fairness with bootstrap uncertainty, selective prediction, and capacity-based Top-*x*% alerting. Several tree-based and boosting models achieved comparable held-out discrimination, while calibrated HGB remained competitive across classification, ranking, and probability-reliability metrics. Threshold choices substantially changed the precision–recall balance, indicating that operating points should be treated as policy choices rather than universal defaults. Fairness audits showed policy-dependent observed group-level differences, especially in alert rates for disability status, although several subgroup error-rate and positive predictive value (PPV) differences remained uncertain. Selective prediction reduced risk on accepted cases as coverage decreased, whereas Top-*x*% alerting fixed outreach volume and made workload–effectiveness trade-offs explicit. Robustness analyses supported the 28-day window as a practical early-warning compromise and showed that absolute PPV values varied across held-out course-presentation splits. The findings suggest that EWS should be evaluated as policy-linked decision systems, integrating model benchmarking, calibration, fairness uncertainty, and capacity-aware decision rules before deployment.

## Introduction

Course withdrawal remains a persistent challenge in digitally mediated higher and distance education. It reduces learning opportunities for students and creates operational pressure for institutions that must allocate limited advising, tutoring, and support resources. Early-warning systems (EWS) have therefore become a prominent application of learning analytics, using digital traces such as virtual learning environment (VLE) interactions, assessment submissions, and activity logs to identify learners who may benefit from timely support [1–3]. In this context, predictive models are valuable not because they produce risk scores in isolation, but because those scores are translated into concrete intervention decisions.

Much of the early-warning literature evaluates models primarily through discrimination metrics such as ROC–AUC or through general classification accuracy [4–6]. These metrics are important, but they do not fully address deployment. In practice, institutions must decide when to trigger alerts, how many learners can be contacted, and how uncertainty should be handled when early-semester evidence is incomplete. A model with acceptable ranking performance may still be poorly calibrated, may produce different subgroup error profiles under different decision rules, or may generate more alerts than available advising capacity can support. Thus, the deployment readiness of an early-warning model depends not only on predictive performance, but also on probability reliability, threshold behavior, fairness under explicit policies, and workload-aware decision design.

This gap between common benchmarking practice and deployment needs becomes especially salient when probabilistic scores are treated as risk estimates. Two models with similar ROC–AUC can rank learners similarly, yet differ substantially in probability reliability. Poor calibration can distort threshold-based alerting and make risk scores difficult to interpret for triage or communication [7,8]. In addition, because EWS outputs can create unequal exposure to false positives and false negatives, subgroup differences in decision rates and error rates should be audited under the actual policies used in deployment [9]. When early evidence is noisy and institutions cannot intervene on every learner, selective prediction provides a principled way to trade coverage for reduced risk by abstaining on lower-confidence cases [10].

This study examines course-withdrawal prediction from this deployment-oriented perspective. Using the Open University Learning Analytics Dataset (OULAD) [11], the analysis constructs early-window features from VLE engagement, assessment-derived signals, and learner background covariates, and evaluates withdrawal prediction under a group-wise split by course presentation. The empirical benchmark covers multiple classifiers, including logistic regression, histogram-based gradient boosting (HGB), random forest, support vector machine, AdaBoost, K-nearest neighbors, XGBoost, LightGBM, and CatBoost. A calibrated HGB model is then retained as the main probabilistic model for downstream policy-oriented analyses because it provides competitive held-out performance and supports calibrated risk scoring.

Beyond model benchmarking, the study evaluates how risk scores behave under operational decision rules. We assess probability reliability using the Brier score, expected calibration error, and reliability diagrams; conduct threshold sensitivity analysis to examine how different operating points affect precision, recall, and workload; and audit fairness across disability status, gender, and age band under both a reference threshold and capacity-based Top-*x*% alerting. To support cautious interpretation, subgroup fairness metrics are reported with bootstrap uncertainty. Confidence-based selective prediction is also implemented to examine the coverage–risk trade-off, distinguishing abstention on uncertain cases from risk-ranking policies that fix outreach capacity.

The contributions of this study are threefold. First, we provide a reproducible evaluation protocol for early-warning models that integrates model benchmarking, calibration assessment, threshold sensitivity, fairness auditing with uncertainty, selective prediction, and capacity-based alerting. Second, using OULAD with an early-semester feature window and group-wise evaluation, we show how deployment-relevant conclusions differ when model outputs are evaluated as inputs to intervention policies rather than as ranking scores alone. Third, we translate predictive performance into intervention-oriented quantities, including alert volume, precision, recall, false-positive exposure, and subgroup alert-rate differences, thereby supporting more transparent early-warning design under limited institutional capacity.

## Literature review

Early-warning systems are best understood as decision-support systems rather than standalone prediction engines. A deployment-oriented review therefore needs to consider not only whether models can rank learners by risk, but also whether their outputs can be interpreted, audited, and translated into feasible intervention policies. We organize the review around four related strands: early-warning systems in learning analytics; predictive model families, benchmarking, and task-aligned evaluation; calibration, fairness, and governance under decision policies; and uncertainty-aware and capacity-aware deployment. We then synthesize these strands to identify the research gap addressed in this study.

### Early-warning systems in learning analytics

EWS have long been a central application of learning analytics. Foundational studies showed that digital learning traces, including VLE activity, assessment information, and engagement indicators, can be used to identify students at risk early enough for institutional support [3]. Institutional deployments further demonstrated that predictive signals can be embedded into advising workflows, where model outputs are not merely reported but operationalized through communication, triage, and support protocols [1,2]. This prediction-to-action perspective clarifies that EWS effectiveness depends not only on model performance, but also on how risk scores are translated into intervention decisions.

Recent work has expanded EWS research beyond single-course demonstrations. Course-agnostic approaches have examined whether models based on learning-platform logs can generalize across courses or institutions, highlighting the importance of representation choices and temporal aggregation in early prediction [12]. Complementary research on predictive errors has shown that false positives and false negatives can reflect unobserved learner circumstances, course design features, or unexpected life events, motivating human judgment and contextual interpretation in EWS implementation [13]. Intervention-oriented studies have also examined whether predictive signals can improve outcomes when coupled with designed support, including personalized interventions and broader learning-analytics intervention frameworks [14,15]. Together, these studies suggest that deployment-ready EWS research should connect prediction quality with actionability, portability, and error consequences.

### Predictive model families, benchmarking, and task-aligned evaluation

A related methodological issue concerns the choice of model families. In learning analytics, early-warning studies have used a wide range of models, from transparent statistical baselines to tree-based ensembles and boosting algorithms [4–6]. Transparent models such as logistic regression remain useful as interpretable baselines, whereas ensemble and boosting methods can capture nonlinearities and interactions in tabular behavioral and assessment data. This motivates benchmarking across multiple model families rather than relying on a single preferred classifier.

This principle is also visible in applied machine-learning research beyond education, where model choice is often matched to data structure and operational purpose. For example, deep recurrent and probabilistic deep-learning approaches have been used for complex energy forecasting and reliability assessment tasks [16,17]. Other studies have applied reinforcement learning or dynamic deep-learning models to predictive management and dynamic value prediction problems in energy systems [18,19]. XGBoost-based and interpretable combinatorial machine-learning approaches have also been used in industrial early-warning and engineering evaluation settings [20,21]. Although these studies address different substantive domains, they illustrate a methodological point relevant to early-warning research: conclusions may depend on the interaction between data representation, model family, and evaluation criterion.

Metric selection must likewise be aligned with the prediction task. In continuous-valued forecasting or regression problems, statistical measures such as correlation-based indices, MAE/MAPE, RMSE, MBE, SI, and related error measures are commonly used; examples include studies of water-pipeline dispersion coefficients and environmental or coastal engineering prediction tasks [22–24]. Course withdrawal prediction, by contrast, is a binary classification and risk-scoring problem. This distinction motivates evaluation frameworks that combine threshold-based classification metrics, ranking metrics, and probability-reliability diagnostics, rather than importing continuous-forecasting error measures without regard to task structure.

### Calibration, fairness, and governance under decision policies

While discrimination metrics such as ROC–AUC measure ranking quality, many real deployments require probabilities that can be interpreted as risk estimates. Calibration concerns whether predicted probabilities correspond to empirical event frequencies, and post-hoc methods such as Platt scaling provide practical mechanisms to adjust probability outputs without changing the underlying classifier [8]. Modern calibration research has shown that high-performing models can be systematically miscalibrated, motivating routine calibration assessment and reporting [7]. In decision contexts, calibration is especially relevant when institutions must set probability thresholds, interpret risk scores for triage, or justify why specific learners are prioritized for outreach [25].

Calibration also interacts with policy choice. Miscalibrated scores can lead to suboptimal thresholding, distorted risk stratification, and inappropriate alert volumes under fixed decision rules or capacity constraints [26–28]. In educational settings, the perceived reliability of probabilistic outputs can influence stakeholder trust and willingness to use learning-analytics recommendations, reinforcing the need for transparent reporting and human oversight [29,30]. Related work on calibrated explanations further emphasizes that uncertainty information and explanation reliability should be considered together when model outputs support decisions [31].

Fairness and governance are equally central when EWS outputs guide student support. In algorithmic fairness, criteria such as equal opportunity motivate auditing whether true positive rates and false positive rates differ across protected groups [9]. In education, such differences may translate into unequal exposure to outreach, false alarms, or missed support opportunities. Education-focused research has therefore emphasized context-specific auditing, data-source awareness, and mitigation strategies across the model life cycle [32,33]. Practitioner-oriented work further stresses intersectionality-aware evaluation and transparent documentation, suggesting that fairness requirements should be treated as institutional governance commitments rather than purely mathematical properties [34]. Human-centred learning analytics and AI research similarly argues for stakeholder agency, safety, trust, and accountability in the design and monitoring of educational AI systems [35]. These perspectives motivate evaluating fairness under the concrete decision policies used in practice, rather than treating fairness as an abstract model-level property.

### Uncertainty-aware and capacity-aware deployment

A practical challenge in early warning is that early-semester evidence is incomplete and noisy, while intervention resources are limited. Selective prediction, also known as classification with abstention or reject option, addresses this by allowing a model to decline predictions on low-confidence cases, thereby trading coverage for reduced risk. Theoretical foundations formalize risk–coverage trade-offs [36], and later work demonstrates selective mechanisms for modern predictive models [10]. Recent surveys further frame abstention as a safety-relevant capability, organizing evaluation around when a model should decline to answer under uncertainty [37].

In EWS deployment, capacity constraints are often handled through fixed decision rules or workload-driven targeting, such as contacting learners above a probability threshold or prioritizing the highest-risk 10–20% of learners. However, learning-analytics studies less often report selective-prediction risk–coverage curves or explicitly connect capacity policies to governance-relevant consequences such as false alarms, missed withdrawals, and subgroup intervention burden. Existing intervention-oriented and workload-aware studies show the importance of connecting risk prediction to feasible support workflows [38,39]. This motivates evaluating both confidence-based abstention and risk-ranking Top-*x*% alerting as complementary decision layers: the former manages uncertainty by withholding low-confidence decisions, while the latter fixes outreach volume and prioritizes the highest predicted risks.

### Research gap

The literature suggests three gaps that are particularly important for deployment-ready early-warning research. First, EWS evaluation often prioritizes discrimination metrics, while probability reliability is less consistently reported despite its direct relevance for threshold-based and capacity-based intervention policies [4,5,7,28]. Second, fairness auditing has gained attention, but subgroup disparities are still often discussed as model-level properties rather than policy-dependent outcomes that should be evaluated under concrete decision rules such as thresholds and Top-*x*% alerting [9,32–34]. Third, uncertainty-aware mechanisms such as selective prediction are well developed in machine learning, but remain underused in learning-analytics deployment studies despite their relevance for balancing intervention workload and misclassification risk [10,36–39].

This study addresses these gaps by proposing a deployment-oriented evaluation protocol for course-withdrawal early warning using OULAD [11]. Specifically, we benchmark multiple model families, assess probability reliability, conduct threshold-sensitive and capacity-based fairness audits with uncertainty intervals, implement selective prediction to quantify coverage–risk trade-offs, and complement the main analysis with robustness checks across observation windows and group-wise evaluation splits. In doing so, the study connects predictive modeling to the policy choices through which early-warning systems are actually deployed.

## Materials and methods

### Dataset, outcome, and evaluation split

We use the Open University Learning Analytics Dataset (OULAD) [11], a publicly available and de-identified secondary dataset that links anonymized learner demographics, assessment records, and time-stamped VLE interaction logs across multiple modules and course presentations. The dataset was accessed for research purposes on 21 February 2026. The authors had no access to information that could identify individual participants during or after data collection. Course offerings are identified by (code_module, code_presentation). At the learner level, studentInfo provides background attributes (e.g., region, highest education, deprivation band, studied credits), and studentRegistration provides course-presentation registration information. For behavioral and performance signals, studentVle records clickstream activity by date and site, and studentAssessment records assessment submissions and scores; these are joined with vle and assessments to obtain activity types and assessment metadata (e.g., due dates and weights) for feature construction.

The prediction target is a binary withdrawal label derived from final_result. Learners with final_result = Withdrawn are coded as positive (*y* = 1), and all other outcomes (Pass/Fail/Distinction) are coded as negative (*y* = 0). Protected attributes (disability, gender, age_band) are excluded from model inputs and retained only for post-hoc fairness auditing, reflecting a deployment-oriented design in which group attributes are used for governance monitoring rather than risk scoring. This design separates predictive modeling from post-hoc governance monitoring and reduces the risk that protected attributes directly drive the risk scores.

To reduce information leakage across offerings and to approximate prospective evaluation, we use a group-wise train–test split by course presentation (code_module × code_presentation). On the primary split, the training set contains *N* = 28,234 learners with withdrawal prevalence 31.93%, and the held-out test set contains *N* = 4,359 learners with withdrawal prevalence 26.20%. This non-trivial class imbalance motivates reporting PR–AUC alongside ROC–AUC, and motivates capacity-aware evaluation that reflects limited outreach resources. We implement the group-wise split using GroupShuffleSplit with test_size = 0.2 and a fixed random seed (random_state = 42). For split-stability robustness checks, we repeat the same group-wise split procedure with seeds 1–5. The primary split is used for all main analyses, whereas the repeated splits are used only to assess split stability across alternative held-out course-presentation partitions.

### Ethics statement

This study used the Open University Learning Analytics Dataset, a publicly available and de-identified secondary dataset. The authors did not recruit human participants, did not collect new data, and had no access to directly identifiable participant information during or after data collection. The Academic Committee of the School of Management, Shenzhen University of Information Technology determined that formal ethical approval was not required because the study involved only secondary analysis of publicly available de-identified data. Informed consent was not obtained by the authors because no new human-participant data were collected and no identifiable private information was accessed.

### Early-window feature construction

We construct predictors using only evidence available in an early observation window. Our primary specification uses the first four weeks of each course presentation (day 0–27), which aligns with common early-warning goals while ensuring that features are computable before mid-course interventions. Feature construction follows two principles: (i) operational availability (features can be computed from routinely logged traces and early assessments), and (ii) leakage prevention (all time-dependent signals are restricted to the early window and post-outcome variables are excluded). For window-sensitivity analyses, the same feature-construction procedure was repeated for 14-day, 28-day, and 42-day windows, with all time-dependent predictors recomputed strictly within the corresponding window.

### VLE engagement features

VLE engagement signals are derived from studentVle, which records daily click counts (sum_click) by learner, date, and site (id_site). We map sites to pedagogical activity categories by joining studentVle with the vle table (via id_site and course-presentation identifiers) to obtain activity_type. For the primary 28-day specification, we restrict all records to 0≤date<28 and aggregate engagement in two complementary ways: (i) activity-type totals, where we sum clicks within each activity type to capture engagement composition; and (ii) weekly trajectories, where we compute weekly click totals by assigning dates to weeks using week=⌊date/7⌋, capturing coarse temporal dynamics such as early drop-off. We also compute total clicks as the sum of weekly totals. Learners with no recorded VLE activity in a category or week receive zeros for the corresponding features, reflecting the operational interpretation of “no observed engagement” during the early window.

### Assessment-derived features

Assessment-based predictors are derived from studentAssessment joined with assessments to obtain due dates and weights. To preserve temporal validity, we restrict to assessments whose due dates fall within the early window (0≤date<28 in the primary specification). For each learner, we compute: (i) the number of assessments due in the window; (ii) an indicator for whether any early assessment submission evidence is available; (iii) early performance summaries including the average normalized score (score/100) and a cumulative weighted score that sums normalized scores weighted by assessment weights; and (iv) timeliness summaries based on lateness, defined as date_submitted minus the assessment due date, summarized by mean and maximum lateness. If a learner has no assessments due in the early window, assessment-derived features are set to zero, encoding “no early assessment evidence” rather than treating such cases as missing at random.

### Leakage prevention and feature availability

All time-dependent predictors are computed strictly within the specified observation window, and the primary analysis uses day 0–27. We exclude post-outcome variables (e.g., unregistration dates) that could introduce label leakage. Protected attributes are never included in the training feature set and are used only for post-hoc auditing. Missing values in background covariates are handled within the modeling pipeline via imputation, ensuring that model training remains faithful to data availability at deployment time.

In addition to early-window VLE and assessment features, we include background covariates from studentInfo as static predictors (e.g., highest education, deprivation band, studied credits, and region), excluding protected attributes. Categorical and numerical covariates are processed as described in subsequent sections.

### Benchmark models, training protocol, and probability calibration

We first benchmarked a set of supervised classifiers for binary withdrawal prediction from the early-window tabular feature set. The benchmark included logistic regression, histogram-based gradient boosting (HGB), random forest (RF), support vector machine (SVM), AdaBoost, K-nearest neighbors (KNN), XGBoost, LightGBM, and CatBoost. These models cover transparent linear classification, distance-based classification, margin-based classification, bagging-based tree ensembles, and gradient-boosting families commonly used for tabular prediction. Because the primary objective of the study is deployment-oriented evaluation rather than exhaustive hyperparameter optimization, all models were trained under a fixed and reproducible protocol, and the held-out group test set was not used for model selection or hyperparameter tuning.

### Preprocessing pipeline

All models were trained within a unified preprocessing pipeline. Categorical covariates were imputed using the most frequent category and one-hot encoded with handle_unknown = ignore to accommodate categories that may appear in the test split but not in training. Numerical features were imputed using the median. For scale-sensitive models, including logistic regression, SVM, and KNN, numerical features were additionally standardized within the training pipeline; tree-based and boosting models used the imputed numerical features without standardization. All preprocessing steps were estimated on the training data only and then applied to the held-out test data. The same feature specification was used for all benchmark models so that performance differences reflected model behavior rather than differences in feature availability or preprocessing.

### Benchmark classifiers and parameter settings

The logistic regression baseline used inverse-frequency class weights, the liblinear solver, and max_iter = 3000. The HGB model used max_iter = 200, learning_rate = 0.05, and max_depth = 6. Random forest was included as a bagging-based tree ensemble with n_estimators = 150, min_samples_leaf = 2, and balanced subsample class weights. SVM was implemented as a linear support vector classifier with C = 1.0, max_iter = 5000, balanced class weights, and sigmoid calibration for probability outputs. AdaBoost was included as an additional boosting baseline with n_estimators = 100 and learning_rate = 0.05. KNN was included as a distance-based nonparametric comparator using n_neighbors = 25 and distance weighting. XGBoost, LightGBM, and CatBoost were included as widely used gradient-boosting implementations for tabular data. XGBoost used n_estimators = 80, learning_rate = 0.05, max_depth = 4, subsample = 0.9, colsample_bytree = 0.9, eval_metric = logloss, and the histogram tree method. LightGBM used n_estimators = 80, learning_rate = 0.05, max_depth = −1, and num_leaves = 31. CatBoost used iterations = 60, learning_rate = 0.05, depth = 6, loss_function = Logloss, and eval_metric = AUC. All settings were fixed before evaluation and were not tuned on the held-out group test set. For the boosting models, we used moderate iteration counts and learning rates to reduce overfitting risk and maintain reproducibility.

These parameter settings were chosen as conservative, reproducible specifications suitable for a model-comparison benchmark rather than as exhaustively optimized configurations. This choice is consistent with the goal of the study: to evaluate deployment-relevant properties of early-warning models, including probability reliability, fairness under explicit decision rules, and capacity-aware decision layers. The held-out group test set was not used for parameter tuning. Full implementation details and code are provided in the accompanying replication materials.

The empirical benchmark was deliberately restricted to models that operate on the same early-window tabular feature representation. Sequence-oriented deep learning architectures, such as CNN, RNN, LSTM, and BiLSTM models, are important alternatives when learner activity is represented as high-frequency temporal sequences or raw event streams. In the present study, however, VLE activity and assessment evidence are represented as engineered early-window aggregates to support transparent auditing, calibration assessment, and policy-oriented interpretation. Including sequence models would therefore introduce a different input representation in addition to a different model family, making the benchmark less directly comparable. We treat sequence-based deep learning models as a natural extension for future work rather than as direct baselines in the present tabular benchmark.

### Post-hoc probability calibration

Because the downstream fairness and capacity-aware analyses require probabilistic risk scores, we retained the HGB model as the main model for post-hoc probability calibration and policy-oriented analyses. For this main model, we applied sigmoid (Platt) calibration as a post-hoc step. Within the training set, we further split the data into a model-fitting subset (80%) and a calibration subset (20%) using a fixed random seed (random_state = 42) and stratification by the outcome. The HGB model was fitted on the model-fitting subset, and a sigmoid calibration mapping was then learned on the calibration subset and applied to transform raw HGB scores into calibrated probabilities. This procedure avoided using test labels for calibration and preserved the integrity of the held-out evaluation.

Throughout, protected attributes (disability, gender, age_band) were not included among predictive features; they were used only after model fitting for fairness auditing. The calibrated HGB model was used as the main probabilistic model for the subsequent threshold-based fairness audits, capacity-based alerting analyses, threshold sensitivity analyses, and selective prediction analyses. Benchmark comparisons across alternative classifiers are reported separately from the calibration and policy analyses.

### Evaluation metrics, fairness auditing, and capacity-aware policies

We adopt a deployment-oriented evaluation perspective in which model outputs are assessed by conventional classification performance, discrimination, probability reliability, group-wise disparities under explicit decision rules, and capacity-aware decision layers. Unless otherwise stated, metrics are computed on the held-out group test split. For model benchmarking, metrics are reported for each candidate classifier. For threshold sensitivity, fairness auditing, selective prediction, and capacity-based policy analyses, we use the calibrated HGB model as the main probabilistic model.

### Classification, discrimination, and calibration metrics

For model benchmarking, we report threshold-based classification metrics at the reference threshold *t* = 0.5, including accuracy, precision, recall, F1 score, and Cohen’s kappa. These metrics summarize the classification behavior induced by a transparent reference rule. Because the positive class is withdrawal and the held-out test prevalence is approximately 26%, we report PR–AUC alongside ROC–AUC. For diagnostic completeness, we also compute the same benchmark metrics on a training-evaluation subset and report them in Supporting Information; all substantive comparisons in the main text are based on the held-out group test split.

ROC–AUC and PR–AUC are reported to evaluate discrimination (ranking quality). Because early-warning interventions often rely on probability thresholds and workload planning, we additionally evaluate probability reliability using the Brier score and expected calibration error (ECE). The Brier score is defined as the mean squared error of predicted probabilities:


$$\begin{array}{c} Brier=\frac{1}{N}\sum_{i=1}^{N}(p_{i}-y_{i}{)}^{2}. \end{array}$$

ECE is computed with *B* = 15 equal-width bins over [0,1]. Let ${ℐ}_{b}=\{i:\;p_{i}\in (\frac{b-1}{B},\frac{b}{B}]\}$ be the index set of bin *b*, and let $conf(b)=\frac{1}{\left|{ℐ}_{b}\right|}\sum_{i\in {ℐ}_{b}}p_{i}$ and $acc(b)=\frac{1}{\left|{ℐ}_{b}\right|}\sum_{i\in {ℐ}_{b}}y_{i}$ denote the mean predicted probability and empirical event frequency in that bin. Then


$$\begin{array}{c} ECE=\sum_{b=1}^{B}\frac{\left|{ℐ}_{b}\right|}{N}\left|acc(b)-conf(b)\right|. \end{array}$$

We complement scalar metrics with reliability diagrams that plot acc(b) against conf(b), enabling a visual diagnosis of over- or under-confidence across the probability range.

### Fairness auditing under a fixed threshold

To assess group-wise disparities under a simple and widely reported decision rule, we use *t* = 0.5 as a transparent reference threshold rather than as a claim that this threshold is universally optimal for deployment. Under this rule, $\hat{y}=1$ if p≥t. For each protected attribute (disability, gender, age_band), we report: (i) the positive prediction rate (PosRate), defined as $\mathrm{Pr}(\hat{y}=1|g)$; (ii) the true positive rate (TPR), defined as $\mathrm{Pr}(\hat{y}=1|y=1,g)$; (iii) the false positive rate (FPR), defined as $\mathrm{Pr}(\hat{y}=1|y=0,g)$; and (iv) positive predictive value (PPV), defined as $\mathrm{Pr}(y=1|\hat{y}=1,g)$. We additionally report disparities relative to the largest group by sample size, providing an interpretable summary of observed group-level differences under the same threshold rule.

### Capacity-based fairness auditing under Top-*x*% alerting

Because institutions often operate under fixed outreach capacity, we also evaluate a capacity-based policy that flags the top *x*% highest-risk learners by predicted probability (risk-ranking). Let k=⌊xN⌋ and let ${𝒮}_{x}$ be the set of indices corresponding to the *k* largest predicted probabilities. The policy predicts $\hat{y}=1$ for $i\in {𝒮}_{x}$ and $\hat{y}=0$ otherwise. For each group, we report the alert count (Alerts), within-group alert rate (Alert rate), TPR, FPR, and positive predictive value (PPV; precision among alerted cases). As above, disparities are reported relative to the largest reference group. This audit makes fairness interpretable as observed differences in intervention burden and error exposure under a realistic capacity constraint.

### Threshold sensitivity analysis

Because operational thresholds are policy choices that depend on advising capacity and error tolerance, we further conducted a threshold sensitivity analysis over t∈{0.3,0.4,0.5,0.6,0.7}. For each threshold, we recomputed accuracy, precision, recall, F1 score, Cohen’s kappa, alert volume, and subgroup fairness metrics. This analysis assesses whether the main deployment-relevant conclusions are specific to the reference threshold or reflect broader precision–recall and intervention-burden trade-offs.

### Bootstrap uncertainty for subgroup fairness metrics

To quantify uncertainty in subgroup fairness estimates, we used nonparametric bootstrap resampling of the held-out test set with 500 resamples. For each resample, subgroup metrics and disparities relative to the largest reference group were recomputed under the same decision rule. We report percentile-based 95% confidence intervals for group-wise rates and disparity estimates. These intervals are intended to support cautious interpretation, especially for smaller subgroups such as learners reporting disability and the 55+ age band.

### Selective prediction (confidence-based abstention)

To operationalize uncertainty management, we implement selective prediction (abstention). We define confidence as $c_{i}=\max(p_{i},1-p_{i})$ and rank cases by $c_{i}$ in descending order. For a given coverage level γ∈(0,1], we accept the top ⌊γN⌋ most confident cases and abstain on the remainder. We report model-centric performance on the accepted subset, including accuracy and risk, where risk=1−accuracy. To connect selective prediction to deployment consequences, we also report intervention-centric quantities computed with respect to the full test set, including alert volume (number of predicted positives among accepted cases at the reference threshold *t* = 0.5), precision (PPV), and overall recall defined as the fraction of all withdrawals in the test set captured among alerted cases. Conceptually, selective prediction (confidence-based abstention) differs from capacity-based alerting (risk-ranking top-*x*%): the former avoids uncertain decisions, whereas *t*he latter fixes workload and prioritizes the highest predicted risks. Reporting both provides complementary policy levers for deployment design.

## Results

### Benchmark model performance

Table 1 reports the held-out test performance of the benchmark models under the primary group-wise split. Several tree-based and boosting models achieved similar discrimination, with ROC–AUC values close to 0.79 and PR–AUC values around 0.66. Random forest had the highest F1 score (0.581), whereas the uncalibrated HGB achieved the highest ROC–AUC (0.796) and a PR–AUC comparable to random forest and LightGBM. XGBoost and CatBoost achieved slightly higher precision at the reference threshold but lower recall, illustrating that threshold-based classification metrics can vary even when ranking metrics are similar.

**Table 1:** Held-out test performance of benchmark models under the primary course-presentation group-wise split.

Model	Accuracy	Precision	Recall	F1	Kappa	ROC–AUC	PR–AUC	Brier	ECE15
Random Forest	0.816	0.720	0.487	0.581	0.469	0.792	0.667	0.145	0.077
HGB (uncalibrated)	0.814	0.721	0.472	0.570	0.458	0.796	0.666	0.140	0.038
LightGBM	0.812	0.711	0.475	0.569	0.455	0.792	0.665	0.141	0.040
HGB (calibrated, sigmoid)	0.814	0.721	0.472	0.570	0.458	0.794	0.664	0.141	0.036
XGBoost	0.815	0.757	0.432	0.550	0.444	0.792	0.662	0.140	0.034
CatBoost	0.816	0.758	0.440	0.557	0.451	0.789	0.656	0.141	0.045
Logistic Regression	0.783	0.597	0.534	0.564	0.420	0.760	0.590	0.166	0.109
KNN	0.801	0.707	0.412	0.521	0.406	0.729	0.589	0.156	0.048
AdaBoost	0.799	0.840	0.289	0.430	0.342	0.747	0.580	0.153	0.055
SVM (linear, calibrated)	0.791	0.741	0.313	0.440	0.336	0.755	0.574	0.173	0.137

Notes: Accuracy, precision, recall, F1, and Cohen’s kappa are computed at the reference threshold *t* = 0.5. ROC–AUC, PR–AUC, Brier score, and ECE15 are computed from the predicted probability scores.

The calibrated HGB model remained competitive across classification, discrimination, and calibration metrics (Accuracy = 0.814, F1 = 0.570, ROC–AUC = 0.794, PR–AUC = 0.664, Brier = 0.141, ECE15 = 0.036). Relative to the prespecified logistic regression baseline, it provided clear gains in ranking performance and probability reliability, while remaining comparable to other tree-based and boosting models. Because the calibrated HGB provided stable probabilistic outputs suitable for downstream policy analyses, we retained it as the main model for threshold sensitivity, fairness auditing, selective prediction, and capacity-based alerting.

Held-out test ROC and precision–recall curves for the benchmark models are provided in Supporting Information (S1 Fig). Training-evaluation metrics and training-stage ROC curves are provided as diagnostic information only in Supporting Information (S1 Table and S2 Fig). Additional diagnostic visualizations for the selected calibrated HGB model, including the confusion-matrix heatmap at the reference threshold, predicted-risk violin plot, and jitter plot of predicted probabilities by observed outcome, are provided in Supporting Information (S3–S5 Figs). Substantive model comparisons are based on the held-out group test results in Table 1; the additional curves and training-stage results are provided as diagnostic supplements.

### Calibration diagnostics

The benchmark results in Table 1 indicate that several tree-based and boosting models achieved similar probability-reliability metrics on the held-out group test set. We therefore use calibration diagnostics not to claim perfect probability estimation, but to assess the practical reliability of the main probabilistic model before using its scores in subsequent threshold-based and capacity-based analyses.

**Fig 1:**
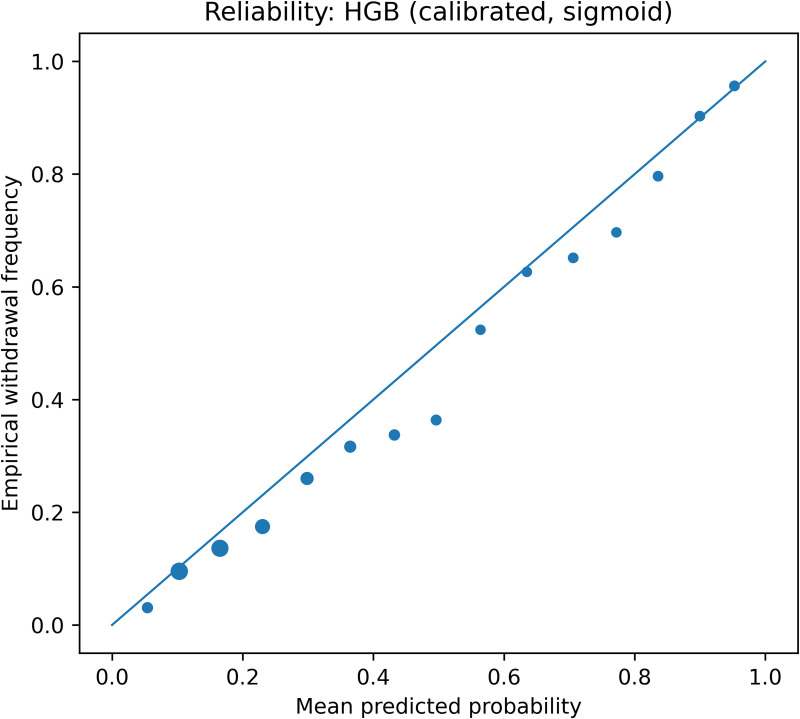
Reliability diagram for the calibrated HGB model on the held-out group test set. The diagonal line represents perfect calibration; deviations from the diagonal indicate over- or under-estimation of withdrawal probabilities in the corresponding probability ranges.

Fig 1 shows the reliability diagram for the calibrated HGB model used in the downstream policy analyses. The curve is broadly aligned with the diagonal, suggesting that predicted probabilities can be interpreted as approximate withdrawal frequencies across score ranges, although some bin-level deviations remain. This diagnostic supports the use of calibrated HGB probabilities as a practical basis for threshold sensitivity analysis, fairness auditing, selective prediction, and capacity-based alerting.

### Threshold sensitivity

Table 2 reports threshold-sensitive classification metrics for the calibrated HGB model. Because ROC–AUC, PR–AUC, Brier score, and ECE15 are computed from the underlying probability scores, they remain unchanged across thresholds; the table therefore focuses on threshold-dependent classification metrics. Lower thresholds increased recall but reduced precision, whereas higher thresholds increased precision at the cost of missed withdrawals. For example, increasing the threshold from 0.30 to 0.70 raised precision from 0.525 to 0.817, while recall decreased from 0.660 to 0.352.

**Table 2:** Threshold sensitivity of the calibrated HGB model on the held-out group test set.

Threshold	Accuracy	Precision	Recall	F1	Kappa
0.30	0.755	0.525	0.660	0.585	0.414
0.40	0.796	0.626	0.547	0.584	0.450
0.50	0.814	0.721	0.472	0.570	0.458
0.60	0.816	0.781	0.415	0.542	0.440
0.70	0.810	0.817	0.352	0.492	0.397

Notes: Metrics are computed by varying the classification threshold applied to the same calibrated probability scores.

The reference threshold *t* = 0.5 produced an intermediate operating profile, with accuracy 0.814, precision 0.721, recall 0.472, F1 score 0.570, and Cohen’s kappa 0.458. These results indicate that *t* = 0.5 should be interpreted as a transparent reference rule for reporting threshold-based metrics rather than as a universally optimal deployment threshold. In practice, the threshold should be selected according to institutional advising capacity, tolerance for false positives, and the cost of missed withdrawals; capacity-based alternatives are examined below. Additional threshold-sensitivity fairness results for disability status are reported in Supporting Information (S2 Table).

### Fairness auditing with bootstrap uncertainty

Table 3 reports the threshold-based fairness audit for disability status at the reference threshold *t* = 0.5, with 95% bootstrap confidence intervals. Disability status showed the most visible point-estimate differences in alert rates. Learners reporting disability had a higher positive prediction rate than non-disabled learners (0.241 vs. 0.165), and the bootstrap interval for the alert-rate difference did not include zero (ΔPosRate = 0.076, 95% CI [0.032, 0.122]). Error-rate differences were less certain: the disabled group had a higher point-estimate FPR (0.086 vs. 0.063) and TPR (0.504 vs. 0.468), but the bootstrap intervals for ΔFPR and ΔTPR included zero. These results should therefore be interpreted as observed group-level differences under the reference threshold rather than causal evidence of discriminatory model behavior.

**Table 3:** Threshold-based fairness audit for disability status at the reference threshold *t* = 0.5.

Group	PosRate	TPR	FPR	PPV
Non-disabled	0.165 [0.154, 0.178]	0.468 [0.434, 0.498]	0.063 [0.055, 0.073]	0.713 [0.677, 0.746]
Disabled	0.241 [0.202, 0.287]	0.504 [0.427, 0.589]	0.086 [0.051, 0.123]	0.775 [0.681, 0.870]
Δ vs. non-disabled	0.076 [0.032, 0.122]	0.036 [−0.050, 0.136]	0.023 [−0.011, 0.061]	0.062 [−0.042, 0.150]

Notes: Values in brackets are bootstrap 95% confidence intervals. PosRate denotes the positive prediction rate under the threshold rule. Δ values are computed relative to the non-disabled group.

Table 4 reports the capacity-based audit for disability status under Top-10% and Top-20% alerting. Under Top-10% alerting, disabled learners had a higher point-estimate alert rate than non-disabled learners (0.130 vs. 0.097), but the bootstrap interval for the alert-rate difference included zero. Under Top-20% alerting, the alert-rate difference was larger and more stable (ΔAlertRate = 0.075, 95% CI [0.030, 0.122]). However, the intervals for differences in TPR, FPR, and PPV generally included zero, indicating uncertainty in subgroup error-rate and precision differences. Complete threshold-based audits for gender and age band are reported in Supporting Information (S3 and S4 Tables), and the corresponding capacity-based audits are reported in Supporting Information (S5 and S6 Tables).

**Table 4:** Capacity-based fairness audit for disability status under Top-10% and Top-20% alerting.

Policy	Group	Alert rate	TPR	FPR	PPV
Top-10%	Non-disabled	0.097 [0.094, 0.100]	0.321 [0.301, 0.338]	0.021 [0.016, 0.027]	0.835 [0.794, 0.877]
Top-10%	Disabled	0.130 [0.095, 0.166]	0.292 [0.218, 0.373]	0.034 [0.013, 0.060]	0.833 [0.717, 0.933]
Top-10%	Δ vs. non-disabled	0.033 [−0.005, 0.072]	−0.029 [−0.114, 0.061]	0.013 [−0.010, 0.041]	−0.001 [−0.123, 0.110]
Top-20%	Non-disabled	0.193 [0.190, 0.197]	0.506 [0.481, 0.530]	0.088 [0.079, 0.097]	0.659 [0.623, 0.697]
Top-20%	Disabled	0.268 [0.228, 0.312]	0.526 [0.449, 0.607]	0.116 [0.074, 0.162]	0.727 [0.638, 0.824]
Top-20%	Δ vs. non-disabled	0.075 [0.030, 0.122]	0.019 [−0.066, 0.111]	0.028 [−0.015, 0.070]	0.068 [−0.022, 0.163]

Notes: Values in brackets are bootstrap 95% confidence intervals. Alert rate is the within-group fraction flagged under the corresponding Top-*x*% capacity rule. PPV denotes precision among alerted learners. Δ values are computed relative to the non-disabled group.

Taken together, the fairness audits show that intervention burden and error profiles depend on the decision rule. The largest point-estimate differences were concentrated in alert rates rather than consistently in TPR, FPR, or PPV, and several subgroup error-rate disparities had wide bootstrap intervals. This uncertainty is important for deployment: subgroup audit results should be monitored as governance signals and interpreted together with sample size, course composition, and the selected intervention policy.

### Selective prediction and capacity-based alerting

**Fig 2:**
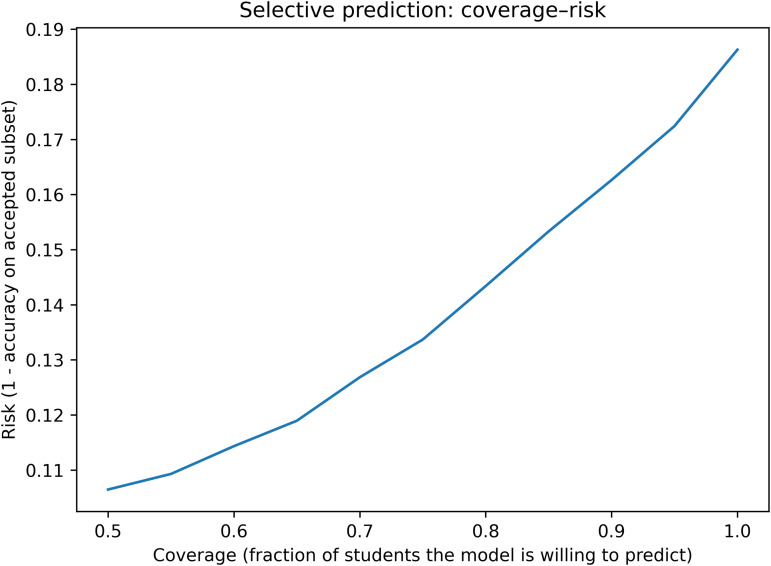
Selective prediction coverage–risk curve for the calibrated HGB model. Coverage denotes the fraction of test cases on which the model makes a prediction after ranking cases by confidence; risk is defined as 1−accuracy on the accepted subset.

Fig 2 and Table 5 summarize two complementary capacity-aware decision layers for the calibrated HGB model. Confidence-based selective prediction reduced risk on the accepted subset as coverage decreased. At full coverage, risk was 0.186; reducing coverage to 80% lowered risk to 0.143, and reducing coverage to 50% lowered risk further to 0.106. This pattern indicates that abstaining on lower-confidence cases can improve the reliability of decisions made on the accepted subset.

**Table 5:** Capacity-aware decision layers for the calibrated HGB model on the held-out group test set. (a) Confidence-based selective prediction. (b) Risk-ranking Top-x% alerting.

Coverage	Accepted *n*	Risk	Alerts	Alert rate	Precision	Recall
1.00	4359	0.186	748	0.172	0.721	0.472
0.80	3487	0.143	506	0.116	0.812	0.360
0.50	2179	0.106	301	0.069	0.894	0.236
**Capacity**	**Alerts**	**Alert rate**	**Precision**	**Recall**	**FPR**	
Top-10%	435	0.100	0.834	0.318	0.022	
Top-20%	871	0.200	0.667	0.509	0.090	
Top-30%	1307	0.300	0.554	0.634	0.181	

Notes: In panel (a), selective prediction applies the reference threshold *t* = 0.5 to accepted cases after ranking learners by prediction confidence. Recall is computed with respect to all withdrawals in the full test set. In panel (b), Top-*x*% alerting ranks all learners by predicted withdrawal risk and fixes the outreach volume at the specified capacity level. Precision is equivalent to positive predictive value among alerted learners.

The intervention-centric consequences show a different trade-off. At full coverage, the reference-threshold rule flagged 748 learners, with precision 0.721 and overall recall 0.472. At 80% coverage, alert volume decreased to 506 learners and precision increased to 0.812, but overall recall decreased to 0.360. At 50% coverage, alerts decreased further to 301 learners and precision increased to 0.894, while overall recall fell to 0.236. Thus, selective prediction can improve precision and reduce workload, but it does so by abstaining from more cases and missing a larger share of withdrawals.

Top-*x*% alerting provides a complementary policy in which the institution fixes outreach capacity and contacts the highest-risk learners. Under Top-10% alerting, the system issued 435 alerts with precision 0.834, recall 0.318, and FPR 0.022. Expanding capacity to Top-20% increased recall to 0.509 but reduced precision to 0.667 and increased FPR to 0.090. At Top-30%, recall increased further to 0.634, while precision declined to 0.554 and FPR increased to 0.181. These results illustrate that selective prediction and capacity-based alerting implement different capacity levers: the former abstains on uncertain cases, whereas the latter fixes outreach volume and prioritizes the highest predicted risks.

### Robustness and sensitivity analyses

We conducted two robustness analyses to assess whether the main findings depended on a single group-wise train–test partition or on the choice of early observation window.

### Split stability across alternative group-wise partitions

Table 6 summarizes the primary split and the five alternative group-wise splits. The primary split used throughout the main analysis, with random seed 42, yielded a Top-10% alerting PPV of 0.834, recall of 0.318, and FPR of 0.022. Across the five alternative group-wise splits using seeds 1–5, Top-10% alerting achieved a mean PPV of 0.896 ± 0.027, recall of 0.327 ± 0.014, and FPR of 0.014 ± 0.004.

**Table 6:** Split-stability summary for the calibrated HGB model under course-presentation group-wise evaluation.

Evaluation	Prevalence	ROC–AUC	PR–AUC	Top-10% PPV	Top-10% recall	Top-10% FPR
Primary split (seed 42)	0.262	0.794	0.664	0.834	0.318	0.022
Alternative splits (seeds 1–5)	0.274 ± 0.012	0.818 ± 0.026	0.724 ± 0.030	0.896 ± 0.027	0.327 ± 0.014	0.014 ± 0.004

Notes: Top-10% metrics refer to capacity-based alerting. The primary split is reported separately because it is the split used for all main analyses. Alternative splits are summarized as mean ± SD across seeds 1–5.

This difference between the primary split and the repeated-split average reflects the structure of group-wise evaluation rather than a change in the alerting definition. Because the split is performed at the course-presentation level, different random seeds hold out different course presentations, leading to variation in test-set prevalence, course composition, and score separability. The primary split is therefore reported separately from the repeated-split summary. Overall, the repeated partitions support the stability of the capacity-based workload–effectiveness pattern, while also showing that absolute PPV values can vary across held-out course-presentation compositions. Detailed split-level results are reported in Supporting Information (S7 Table).

### Window sensitivity with bootstrap uncertainty

Table 7 summarizes point estimates for the window-sensitivity analysis, with bootstrap 95% confidence intervals reported in Supporting Information (S8 Table). Expanding the observation window from 14 days to 28 days substantially improved discrimination and classification performance: ROC–AUC increased from 0.727 to 0.794, PR–AUC increased from 0.556 to 0.664, and F1 increased from 0.441 to 0.570. Extending the window further to 42 days produced only modest additional gains in discrimination (ROC–AUC = 0.804; PR–AUC = 0.675), although F1 increased to 0.593. Probability reliability remained broadly stable, with Brier scores decreasing from 0.162 at 14 days to 0.141 at 28 days and 0.139 at 42 days, while ECE15 remained within a narrow range of 0.026–0.038.

**Table 7:** Window-sensitivity summary for the calibrated HGB model.

Window	Accuracy	F1	ROC–AUC	PR–AUC	Brier	ECE15
14d (2w)	0.780	0.441	0.727	0.556	0.162	0.026
28d (4w)	0.814	0.570	0.794	0.664	0.141	0.036
42d (6w)	0.811	0.593	0.804	0.675	0.139	0.038

Notes: Values are point estimates on the held-out group test set. Accuracy and F1 are computed at the reference threshold *t* = 0.5. Bootstrap 95% confidence intervals are reported in Supporting Information (S8 Table).

These results support the 28-day window as a practical early-warning compromise. It captures most of the predictive gain obtained by extending beyond the first two weeks, while preserving earlier intervention timing than a six-week window. The window-sensitivity results also indicate that the main deployment-oriented conclusions are not artifacts of a single observation-window specification.

## Discussion

This study evaluated course-withdrawal early-warning models from a deployment-oriented perspective, emphasizing that discrimination alone is insufficient once predicted scores are converted into intervention policies. The findings highlight three implications for the deployment of early-warning models. First, model benchmarking showed that several tree-based and boosting models achieved comparable held-out discrimination, suggesting that model choice should not be reduced to a single ranking metric. Second, the threshold, fairness, selective-prediction, and capacity-alerting analyses demonstrate that the same probabilistic scores can lead to different intervention consequences depending on the decision rule. Third, the robustness analyses indicate that the main workload–effectiveness patterns are broadly stable across observation windows and alternative group-wise splits, although absolute PPV values can vary across held-out course-presentation compositions. Together, these findings support the central argument that early-warning models should be evaluated as policy-linked decision systems rather than as stand-alone classifiers.

The model-benchmarking results qualify the interpretation of “best model” selection. Random forest achieved the highest F1 score, and several boosting models produced similar ROC–AUC and PR–AUC values; thus, the calibrated HGB should not be interpreted as uniformly superior across all metrics. Its value in this study lies in its combination of competitive discrimination, acceptable probability reliability, and suitability for downstream threshold-, fairness-, and capacity-based analyses. This distinction is important for early-warning deployment: institutions do not act on rankings alone, but must choose thresholds, outreach capacities, and triage rules. The threshold-sensitivity analysis shows that lower thresholds improve recall at the cost of precision, while higher thresholds improve precision but miss more withdrawals. Accordingly, *t* = 0.5 should be treated as a transparent reference rule rather than as a universal operating point. In practice, threshold selection should be jointly determined by advising capacity, the expected harm of missed withdrawals, the burden of false alarms, and the level of human review available for borderline cases.

The fairness findings should be interpreted as policy-dependent observed group-level differences rather than causal evidence of discriminatory model behavior. This distinction is important because protected attributes were excluded from model inputs and were used only for post-hoc auditing. In the main disability-status audit, learners reporting disability had a higher alert rate under the reference threshold, and the bootstrap interval for the positive-prediction-rate difference did not include zero. However, the bootstrap intervals for several error-rate and PPV differences included zero, indicating that subgroup error profiles should be interpreted with caution rather than as stable causal effects. Under capacity-based alerting, the Top-20% policy showed a more stable alert-rate difference for disability status, whereas differences in TPR, FPR, and PPV remained uncertain. These findings suggest that fairness monitoring should be embedded in the decision policy itself: whenever a threshold or Top-*x*% capacity rule is selected, institutions should report subgroup alert rates, TPR, FPR, and PPV with uncertainty intervals, and use the results as governance signals for review rather than as definitive proof of discrimination.

The capacity-aware analyses show that prediction quality and intervention workload cannot be separated. Confidence-based selective prediction reduced risk on the accepted subset and increased precision as coverage decreased, but it also reduced overall recall because more cases were abstained. Top-*x*% alerting implemented a different policy lever: it fixed the number of learners contacted and prioritized the highest predicted risks, increasing recall as capacity expanded while lowering precision and increasing false-positive exposure. These two decision layers support different institutional needs. Selective prediction is useful when the institution wants to avoid uncertain automated decisions and route ambiguous cases to human review or lower-intensity support. Top-*x*% alerting is useful when the institution has a fixed advising capacity and must decide how many learners can be contacted. In practice, the two can be combined into a tiered workflow: high-risk and high-confidence learners receive direct outreach, medium-risk or lower-confidence learners receive lighter-touch support or human review, and all policies are periodically audited for calibration, workload, and subgroup effects.

The robustness analyses further clarify the stability and limits of these findings. The split-stability analysis explains why the primary-split Top-10% PPV differs from the repeated-split average: group-wise splitting holds out different course presentations, so test-set prevalence, course composition, and score separability vary across seeds. The primary split produced a Top-10% PPV of 0.834, whereas the five alternative group-wise splits produced a higher repeated-split average of 0.896 ± 0.027. This discrepancy should therefore be understood as split-composition sensitivity under course-presentation-level evaluation, not as a change in the alerting definition. The window-sensitivity analysis also supports the 28-day observation window as a practical compromise. Moving from 14 to 28 days produced a substantial gain in discrimination and F1 score, whereas extending to 42 days produced only modest additional discrimination gains while delaying potential intervention. These robustness checks reinforce the central deployment message: early-warning performance should be evaluated not only as a single held-out score, but as a set of policy-relevant trade-offs that may shift with course composition, observation window, and institutional capacity.

## Conclusion

This study examined course-withdrawal early-warning modeling as a deployment-oriented decision problem rather than as a stand-alone prediction task. Using OULAD and an early-window feature specification, the analysis combined multi-model benchmarking with calibration assessment, threshold sensitivity, fairness auditing, selective prediction, and capacity-based alerting. The central implication is that early-warning evaluation should not stop at discrimination or model ranking: predicted scores must also be assessed for whether they can support reliable, interpretable, and auditable intervention decisions under realistic institutional constraints.

The policy analyses show that deployment choices are not merely technical details. Thresholds, capacity rules, and abstention mechanisms reshape precision, recall, alert volume, and subgroup exposure. For this reason, early-warning systems should report not only aggregate predictive performance, but also the consequences of the decision rules through which risk scores are operationalized. Fairness results should likewise be interpreted as governance signals under specific policies, especially when subgroup estimates are uncertain or based on relatively small samples.

Capacity-aware analyses and robustness checks add a workload-sensitive perspective to early-warning deployment. Selective prediction and Top-*x*% alerting provide complementary ways to manage limited advising resources: the former reduces automated decisions on uncertain cases, while the latter fixes outreach volume by prioritizing the highest predicted risks. The robustness analyses also show that practical conclusions depend on observation timing and held-out course composition. The 28-day window offered a useful balance between early intervention and predictive gain, but variation across group-wise splits indicates that deployment decisions should be validated under the course structures and resource constraints of the target institution.

Future work should extend this protocol in several directions. First, external validation across additional institutions, platforms, and course structures is needed to examine whether calibration, subgroup patterns, and capacity trade-offs remain stable beyond OULAD. Second, fairness auditing should be expanded to intersectional and small-subgroup settings, using uncertainty intervals and, where possible, multi-term aggregation to avoid over-interpreting sparse subgroup estimates. Third, future studies should evaluate alternative temporal representations, including sequence-based models for raw event streams, and compare them with transparent tabular early-warning pipelines under the same deployment-oriented criteria. Finally, prospective or quasi-experimental studies are needed to connect decision-layer design with actual intervention effectiveness, including student outcomes, advising workload, perceived legitimacy, and unintended consequences of risk-based outreach.

## Supporting information

S1 Table Training-evaluation performance of benchmark models. Notes: Metrics are computed on a training-evaluation subset and are provided as diagnostic information only. Substantive model comparisons in the main text are based on the held-out course-presentation group test split.
(PDF)

S1 Fig Held-out test (A) ROC and (B) precision–recall curves for the benchmark models. (TIF)

S2 Fig Training-evaluation ROC curves for the benchmark models. Notes: These curves are included only as training-stage diagnostics; held-out test performance is used for substantive model comparison.
(TIF)

S2 Table Threshold-sensitivity fairness audit for disability status. Notes: Entries report point-estimate differences for disabled learners relative to non-disabled learners across reference thresholds.
(PDF)

S3 Table Threshold-based fairness audit by gender. Notes: Metrics are computed at the reference threshold *t* = 0.5. Values in brackets are bootstrap 95% confidence intervals. The reference group is M.
(PDF)

S4 Table Threshold-based fairness audit by age band. Notes: Metrics are computed at the reference threshold *t* = 0.5. Values in brackets are bootstrap 95% confidence intervals. The reference group is 0–35.
(PDF)

S5 Table Capacity-based fairness audit by gender. Notes: Metrics are computed under Top-10%, Top-20%, and Top-30% alerting policies. Values in brackets are bootstrap 95% confidence intervals. Alert rate is the within-group fraction flagged under the corresponding capacity rule. The reference group is M.
(PDF)

S6 Table Capacity-based fairness audit by age band. Notes: Metrics are computed under Top-10%, Top-20%, and Top-30% alerting policies. Values in brackets are bootstrap 95% confidence intervals. Alert rate is the within-group fraction flagged under the corresponding capacity rule. The reference group is 0–35. Some intervals for the 55+ group may appear degenerate because very few cases were alerted under the corresponding capacity rule.
(PDF)

S7 Table Detailed split-stability results. Notes: Results are reported for the primary split and five alternative course-presentation group-wise splits. Top-10% metrics refer to capacity-based alerting using the calibrated HGB model.
(PDF)

S8 Table Window sensitivity with bootstrap 95% confidence intervals. Notes: Results are reported for the calibrated HGB model. All time-dependent features were recomputed within the corresponding observation window.
(PDF)

S3 Fig Confusion-matrix heatmap for the calibrated HGB model at the reference threshold *t* = 0.5 on the held-out group test set. (TIF)

S4 Fig Violin plot of predicted withdrawal probabilities by observed outcome on the held-out group test set. (TIF)

S5 Fig Jitter plot of predicted withdrawal probabilities by observed outcome on the held-out group test set. (TIF)
