# Correction: Calibrated early-warning models with fairness auditing and selective prediction for course withdrawal risk: Evidence from OULAD

**DOI:** 10.1371/journal.pone.0355736

**Published:** 2026-08-07

**Authors:** Suhan Wu, Jingyi Duan, Min Luo

S3 Fig was uploaded incorrectly. Please see the correct S3 Fig here.

## Supporting information

S3 Fig Confusion-matrix heatmap for the calibrated HGB model at the reference threshold t = 0.5 on the held-out group test set. (TIF)
