# Supplementary material for: Calibrated early-warning models with fairness auditing and selective prediction for course withdrawal risk: Evidence from OULAD
Source: PLoS One. 2026 Jul 15;21(7):e0352867. doi: 10.1371/journal.pone.0352867 (PMC13372148; doi:10.1371/journal.pone.0352867)
Supplement: S1 Table — Notes: Metrics are computed on a training-evaluation subset and are provided as diagnostic information only. Substantive model comparisons in the main text are based on the held-out course-presentation group test split. (PDF) [file pone.0352867.s001.pdf]

S1 Table. Training-evaluation performance of benchmark models.

Model	Accuracy	Precision	Recall	F1	Kappa	ROC–AUC	PR–AUC	Brier	ECE15
Random Forest	0.976	0.946	0.980	0.963	0.946	0.991	0.976	0.047	0.137
HGB (uncalibrated)	0.828	0.824	0.575	0.677	0.565	0.866	0.794	0.127	0.026
LightGBM	0.828	0.827	0.570	0.675	0.563	0.863	0.792	0.128	0.028
HGB (calibrated, sigmoid)	0.828	0.814	0.585	0.681	0.567	0.864	0.793	0.127	0.016
XGBoost	0.817	0.817	0.534	0.646	0.529	0.842	0.768	0.135	0.025
CatBoost	0.814	0.813	0.527	0.640	0.522	0.836	0.761	0.138	0.031
Logistic Regression	0.788	0.663	0.661	0.662	0.508	0.820	0.726	0.160	0.123
KNN	1.000	0.999	0.999	0.999	0.999	1.000	1.000	0.000	0.000
AdaBoost	0.767	0.771	0.364	0.495	0.367	0.767	0.653	0.167	0.061
SVM (linear, calibrated)	0.765	0.874	0.292	0.438	0.334	0.777	0.697	0.177	0.138

Notes: Metrics are computed on a training-evaluation subset and are provided as diagnostic information only. Substantive model comparisons in the main text are based on the held-out course-presentation group test split.
