# Supplementary figures and images for: Calibrated early-warning models with fairness auditing and selective prediction for course withdrawal risk: Evidence from OULAD
Source: PLoS One. 2026 Jul 15;21(7):e0352867. doi: 10.1371/journal.pone.0352867 (PMC13372148; doi:10.1371/journal.pone.0352867)

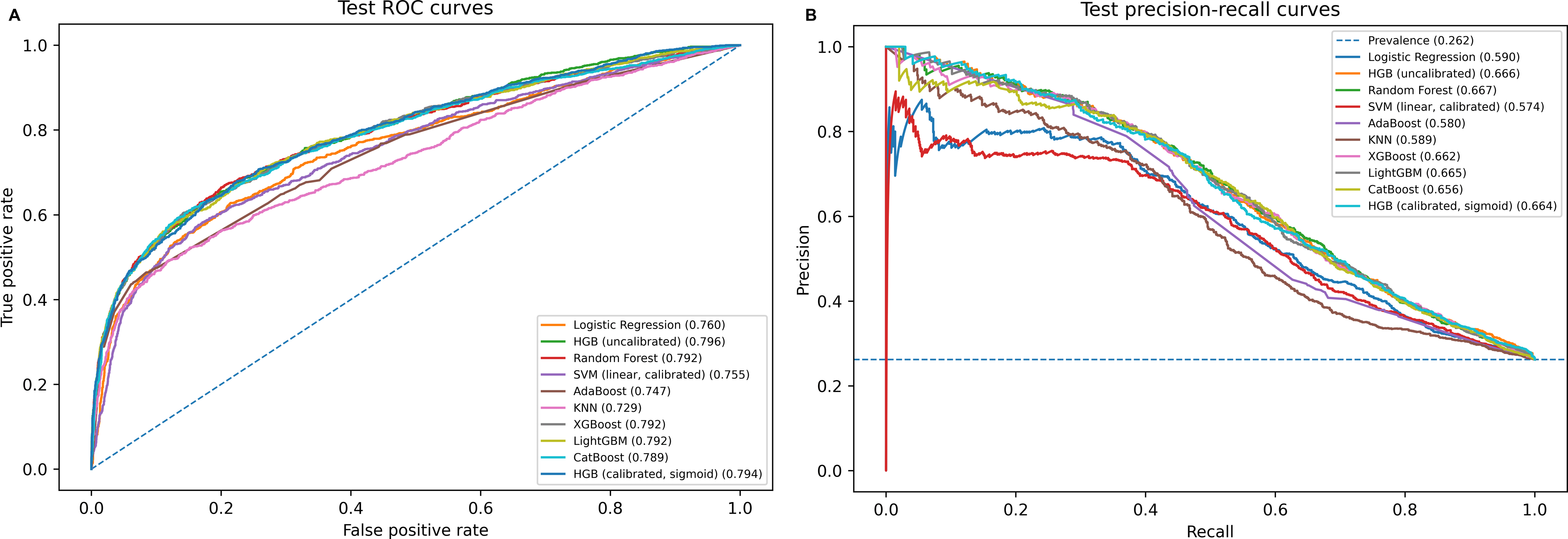

Supplement: S1 Fig — (TIF) [file pone.0352867.s002.tif]

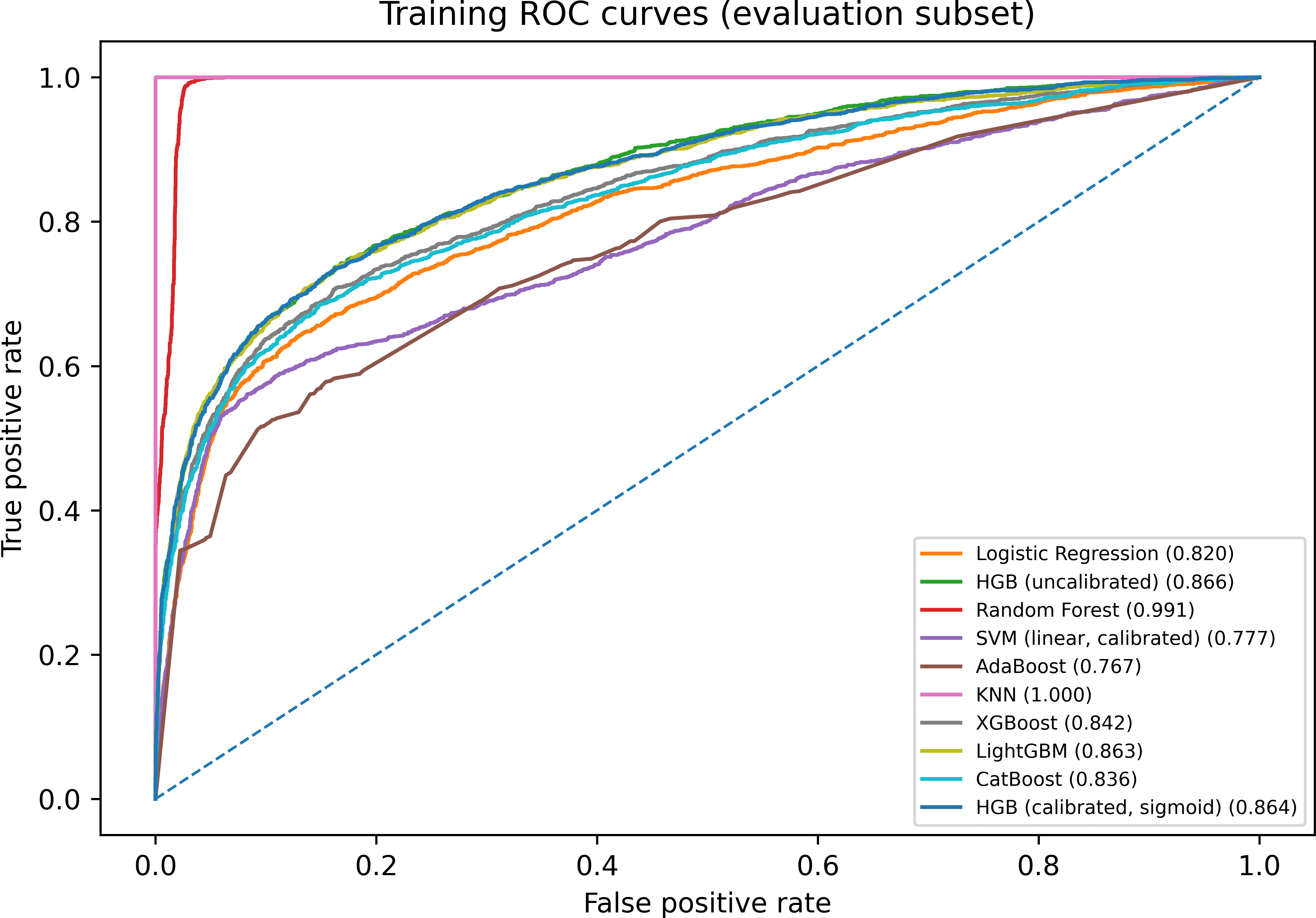

Supplement: S2 Fig — Notes: These curves are included only as training-stage diagnostics; held-out test performance is used for substantive model comparison. (TIF) [file pone.0352867.s003.tif]

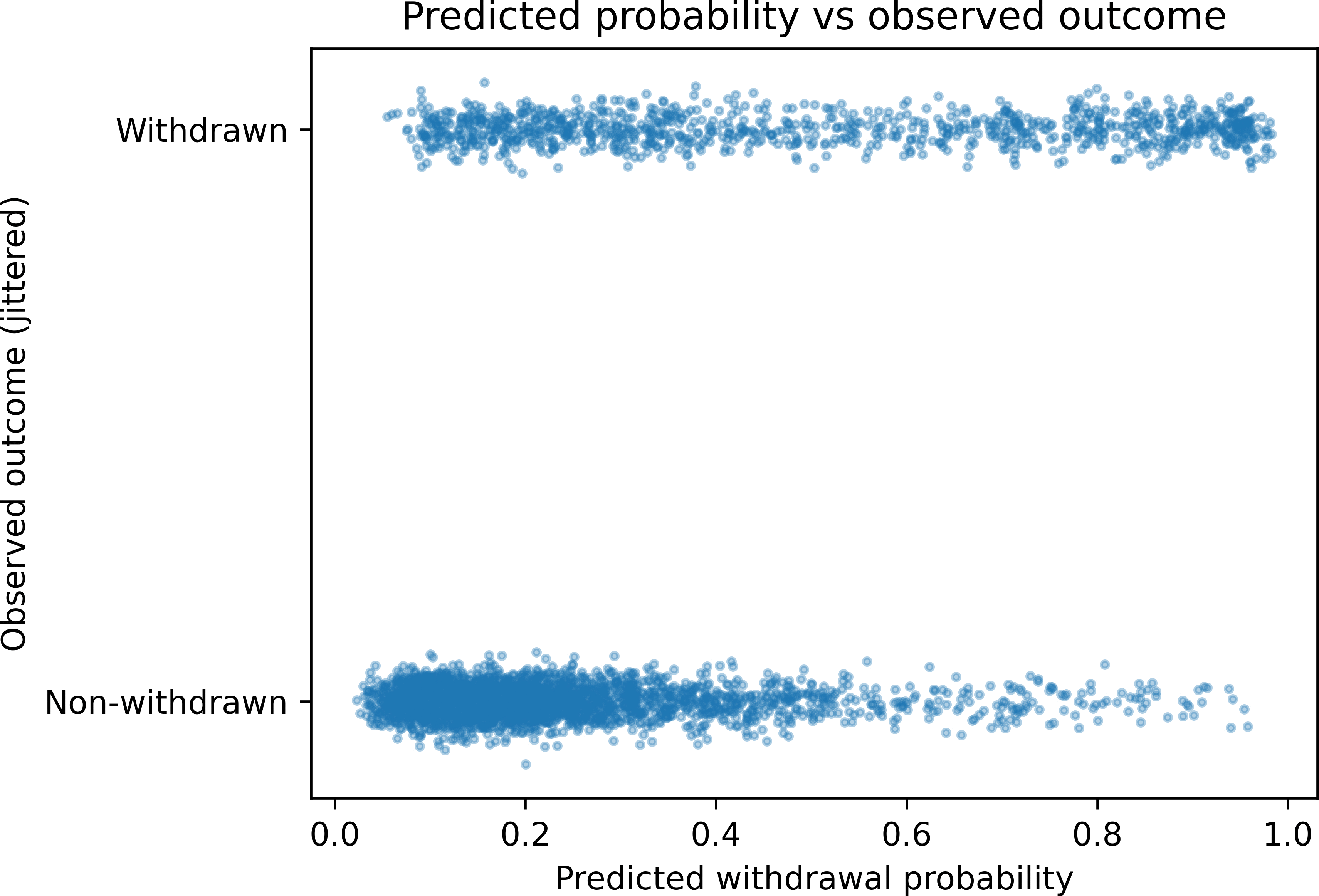

Supplement: S3 Fig — (TIF) [file pone.0352867.s011.tif]

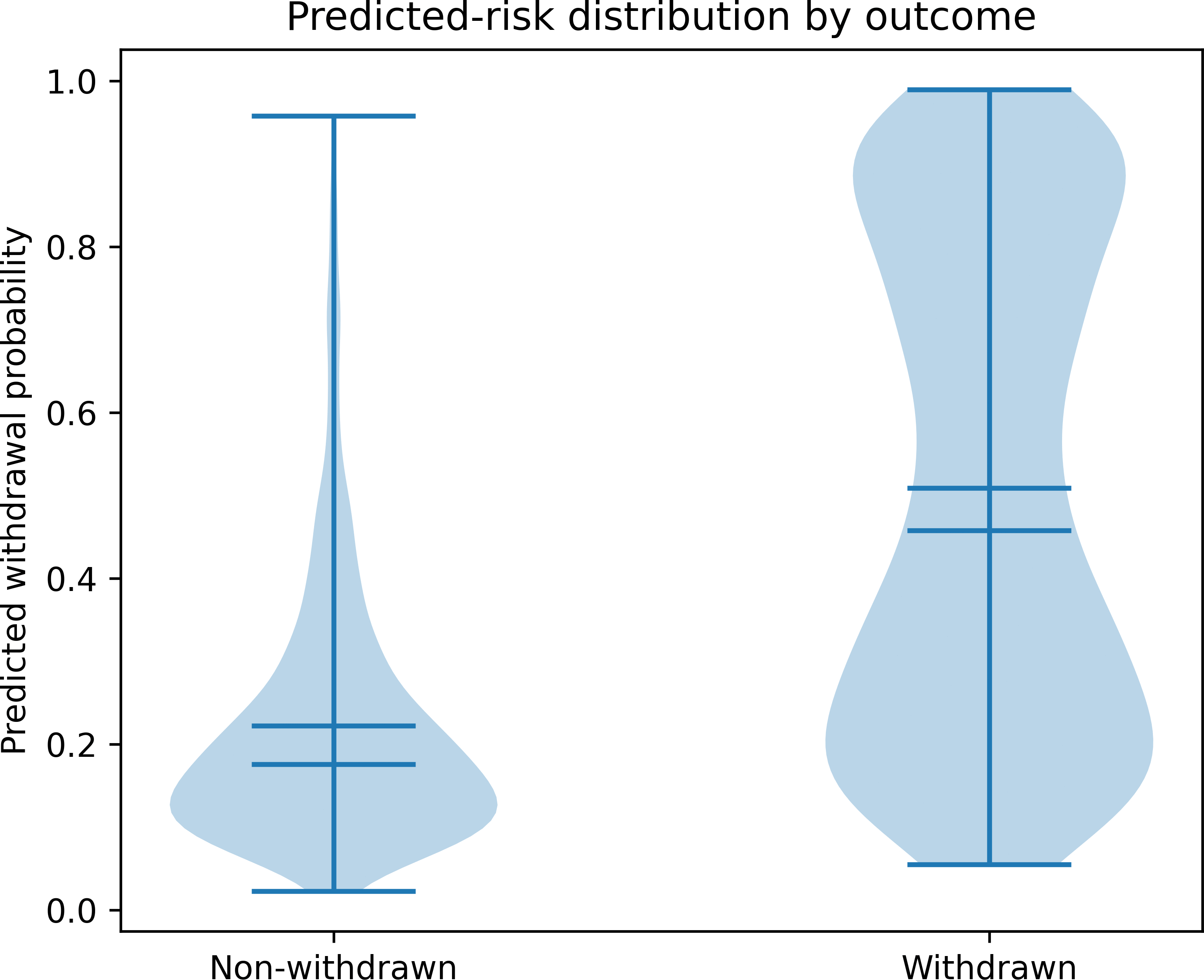

Supplement: S4 Fig — (TIF) [file pone.0352867.s012.tif]
