# Supplementary material for: Calibrated early-warning models with fairness auditing and selective prediction for course withdrawal risk: Evidence from OULAD
Source: PLoS One. 2026 Jul 15;21(7):e0352867. doi: 10.1371/journal.pone.0352867 (PMC13372148; doi:10.1371/journal.pone.0352867)
Supplement: S2 Table — Notes: Entries report point-estimate differences for disabled learners relative to non-disabled learners across reference thresholds. (PDF) [file pone.0352867.s004.pdf]

S2 Table. Threshold-sensitivity fairness audit for disability status.

Threshold	ΔPosRate	ΔTPR	ΔFPR	ΔPPV
0.30	0.090	0.013	0.051	0.089
0.40	0.081	0.033	0.028	0.089
0.50	0.076	0.036	0.023	0.062
0.60	0.046	-0.015	0.011	0.045
0.70	0.028	-0.043	0.007	0.029

Notes: Entries report point-estimate differences for disabled learners relative to non-disabled learners across reference thresholds.
