# Supplementary material for: Calibrated early-warning models with fairness auditing and selective prediction for course withdrawal risk: Evidence from OULAD
Source: PLoS One. 2026 Jul 15;21(7):e0352867. doi: 10.1371/journal.pone.0352867 (PMC13372148; doi:10.1371/journal.pone.0352867)
Supplement: S3 Table — Notes: Metrics are computed at the reference threshold t = 0.5. Values in brackets are bootstrap 95% confidence intervals. The reference group is M. (PDF) [file pone.0352867.s005.pdf]

S3 Table. Threshold-based fairness audit by gender.

Group	n	PosRate	TPR	FPR	PPV
M	3139	0.170 [0.156, 0.185]	0.477 [0.442, 0.510]	0.070 [0.060, 0.080]	0.692 [0.655, 0.730]
F	1220	0.175 [0.155, 0.196]	0.460 [0.410, 0.509]	0.052 [0.038, 0.069]	0.793 [0.737, 0.847]
Δ F vs. M	–	0.004 [-0.019, 0.029]	-0.017 [-0.071, 0.042]	-0.018 [-0.035, -0.000]	0.102 [0.038, 0.166]

Notes: Metrics are computed at the reference threshold $t = 0.5$. Values in brackets are bootstrap 95% confidence intervals. The reference group is M.
