# Supplementary material for: Calibrated early-warning models with fairness auditing and selective prediction for course withdrawal risk: Evidence from OULAD
Source: PLoS One. 2026 Jul 15;21(7):e0352867. doi: 10.1371/journal.pone.0352867 (PMC13372148; doi:10.1371/journal.pone.0352867)
Supplement: S4 Table — Notes: Metrics are computed at the reference threshold t = 0.5. Values in brackets are bootstrap 95% confidence intervals. The reference group is 0–35. (PDF) [file pone.0352867.s006.pdf]

S4 Table. Threshold-based fairness audit by age band.

Group	n	PosRate	TPR	FPR	PPV
0–35	2939	0.181 [0.168, 0.196]	0.489 [0.454, 0.522]	0.069 [0.059, 0.080]	0.722 [0.682, 0.761]
35–55	1352	0.152 [0.133, 0.174]	0.432 [0.382, 0.492]	0.058 [0.045, 0.074]	0.714 [0.651, 0.772]
55+	68	0.147 [0.068, 0.234]	0.500 [0.244, 0.769]	0.038 [0.000, 0.098]	0.800 [0.545, 1.000]
Δ 35–55 vs. 0–35	–	-0.029 [-0.051, -0.004]	-0.056 [-0.116, 0.017]	-0.010 [-0.028, 0.007]	-0.008 [-0.080, 0.064]
Δ 55+ vs. 0–35	–	-0.034 [-0.111, 0.047]	0.011 [-0.245, 0.294]	-0.030 [-0.074, 0.030]	0.078 [-0.184, 0.297]

Notes: Metrics are computed at the reference threshold $t = 0.5$. Values in brackets are bootstrap 95% confidence intervals. The reference group is 0–35.
