# Supplementary material for: Calibrated early-warning models with fairness auditing and selective prediction for course withdrawal risk: Evidence from OULAD
Source: PLoS One. 2026 Jul 15;21(7):e0352867. doi: 10.1371/journal.pone.0352867 (PMC13372148; doi:10.1371/journal.pone.0352867)
Supplement: S5 Table — Notes: Metrics are computed under Top-10%, Top-20%, and Top-30% alerting policies. Values in brackets are bootstrap 95% confidence intervals. Alert rate is the within-group fraction flagged under the corresponding capacity rule. The reference group is M. (PDF) [file pone.0352867.s007.pdf]

S5 Table. Capacity-based fairness audit by gender.

Policy	Group	Alert Rate	TPR	FPR	PPV
Top-10%	M	0.101 [0.095, 0.106]	0.334 [0.309, 0.358]	0.024 [0.017, 0.030]	0.820 [0.774, 0.871]
Top-10%	F	0.098 [0.084, 0.111]	0.283 [0.244, 0.321]	0.018 [0.009, 0.027]	0.874 [0.810, 0.934]
Top-10%	Δ F vs. M	-0.003 [-0.022, 0.015]	-0.051 [-0.107, 0.003]	-0.007 [-0.017, 0.005]	0.054 [-0.016, 0.131]
Top-20%	M	0.198 [0.190, 0.204]	0.514 [0.486, 0.543]	0.094 [0.083, 0.104]	0.642 [0.606, 0.684]
Top-20%	F	0.206 [0.188, 0.225]	0.499 [0.456, 0.547]	0.080 [0.063, 0.096]	0.729 [0.673, 0.785]
Top-20%	Δ F vs. M	0.008 [-0.016, 0.035]	-0.015 [-0.068, 0.047]	-0.014 [-0.035, 0.007]	0.087 [0.023, 0.150]
Top-30%	M	0.295 [0.286, 0.303]	0.631 [0.597, 0.655]	0.185 [0.172, 0.196]	0.528 [0.498, 0.561]
Top-30%	F	0.311 [0.293, 0.336]	0.640 [0.593, 0.685]	0.170 [0.148, 0.199]	0.618 [0.563, 0.673]
Top-30%	Δ F vs. M	0.016 [-0.010, 0.051]	0.009 [-0.046, 0.071]	-0.015 [-0.040, 0.020]	0.091 [0.027, 0.148]

Notes: Metrics are computed under Top-10%, Top-20%, and Top-30% alerting policies. Values in brackets are bootstrap 95% confidence intervals. Alert rate is the within-group fraction flagged under the corresponding capacity rule. The reference group is M.
