# Supplementary material for: Calibrated early-warning models with fairness auditing and selective prediction for course withdrawal risk: Evidence from OULAD
Source: PLoS One. 2026 Jul 15;21(7):e0352867. doi: 10.1371/journal.pone.0352867 (PMC13372148; doi:10.1371/journal.pone.0352867)
Supplement: S6 Table — Notes: Metrics are computed under Top-10%, Top-20%, and Top-30% alerting policies. Values in brackets are bootstrap 95% confidence intervals. Alert rate is the within-group fraction flagged under the corresponding capacity rule. The reference group is 0–35. Some intervals for the 55+ group may appear degenerate because very few cases were alerted under the corresponding capacity rule. (PDF) [file pone.0352867.s008.pdf]

S6 Table. Capacity-based fairness audit by age band.

Policy	Group	Alert Rate	TPR	FPR	PPV
Top-10%	0–35	0.104 [0.098, 0.110]	0.324 [0.301, 0.349]	0.024 [0.016, 0.030]	0.833 [0.782, 0.884]
Top-10%	35–55	0.090 [0.077, 0.103]	0.297 [0.252, 0.344]	0.021 [0.012, 0.029]	0.828 [0.760, 0.896]
Top-10%	55+	0.103 [0.037, 0.172]	0.438 [0.200, 0.700]	0.000 [0.000, 0.000]	1.000 [1.000, 1.000]
Top-10%	Δ 35–55 vs. 0–35	-0.014 [-0.033, 0.005]	-0.027 [-0.087, 0.037]	-0.003 [-0.015, 0.009]	-0.005 [-0.086, 0.080]
Top-10%	Δ 55+ vs. 0–35	-0.001 [-0.069, 0.071]	0.113 [-0.129, 0.377]	-0.024 [-0.030, -0.016]	0.167 [0.116, 0.218]
Top-20%	0–35	0.214 [0.205, 0.221]	0.529 [0.501, 0.555]	0.099 [0.086, 0.110]	0.660 [0.622, 0.708]
Top-20%	35–55	0.171 [0.156, 0.191]	0.462 [0.415, 0.519]	0.073 [0.059, 0.090]	0.680 [0.620, 0.741]
Top-20%	55+	0.147 [0.072, 0.238]	0.500 [0.244, 0.769]	0.038 [0.000, 0.114]	0.800 [0.500, 1.000]
Top-20%	Δ 35–55 vs. 0–35	-0.044 [-0.065, -0.014]	-0.067 [-0.131, 0.009]	-0.026 [-0.045, -0.003]	0.019 [-0.058, 0.089]
Top-20%	Δ 55+ vs. 0–35	-0.067 [-0.140, 0.028]	-0.029 [-0.287, 0.243]	-0.061 [-0.099, 0.012]	0.140 [-0.180, 0.338]
Top-30%	0–35	0.323 [0.313, 0.331]	0.654 [0.623, 0.679]	0.202 [0.187, 0.215]	0.542 [0.510, 0.578]
Top-30%	35–55	0.253 [0.235, 0.273]	0.591 [0.541, 0.638]	0.139 [0.123, 0.161]	0.588 [0.531, 0.641]
Top-30%	55+	0.235 [0.142, 0.339]	0.562 [0.333, 0.844]	0.135 [0.044, 0.235]	0.562 [0.333, 0.800]
Top-30%	Δ 35–55 vs. 0–35	-0.070 [-0.096, -0.041]	-0.063 [-0.127, 0.000]	-0.063 [-0.087, -0.033]	0.046 [-0.020, 0.102]
Top-30%	Δ 55+ vs. 0–35	-0.088 [-0.184, 0.020]	-0.091 [-0.330, 0.181]	-0.067 [-0.156, 0.036]	0.021 [-0.214, 0.266]

Notes: Metrics are computed under Top-10%, Top-20%, and Top-30% alerting policies. Values in brackets are bootstrap 95% confidence intervals. Alert rate is the within-group fraction flagged under the corresponding capacity rule. The reference group is 0–35. Some intervals for the 55+ group may appear degenerate because very few cases were alerted under the corresponding capacity rule.
