# Supplementary material for: Calibrated early-warning models with fairness auditing and selective prediction for course withdrawal risk: Evidence from OULAD
Source: PLoS One. 2026 Jul 15;21(7):e0352867. doi: 10.1371/journal.pone.0352867 (PMC13372148; doi:10.1371/journal.pone.0352867)
Supplement: S7 Table — Notes: Results are reported for the primary split and five alternative course-presentation group-wise splits. Top-10% metrics refer to capacity-based alerting using the calibrated HGB model. (PDF) [file pone.0352867.s009.pdf]

S7 Table. Detailed split-stability results.

Split	Test N	Prev.	ROC–AUC	PR–AUC	Top-10% PPV	Top-10% recall	Top-10% FPR
Primary	4359	0.262	0.794	0.664	0.834	0.318	0.022
1	8531	0.283	0.822	0.751	0.940	0.332	0.008
2	5511	0.285	0.845	0.738	0.877	0.307	0.017
3	5942	0.256	0.790	0.683	0.872	0.341	0.017
4	6341	0.279	0.842	0.747	0.890	0.319	0.015
5	7417	0.268	0.793	0.701	0.903	0.337	0.013

Notes: Results are reported for the primary split and five alternative course-presentation group-wise splits. Top-10% metrics refer to capacity-based alerting using the calibrated HGB model.
