# Supplementary material for: Calibrated early-warning models with fairness auditing and selective prediction for course withdrawal risk: Evidence from OULAD
Source: PLoS One. 2026 Jul 15;21(7):e0352867. doi: 10.1371/journal.pone.0352867 (PMC13372148; doi:10.1371/journal.pone.0352867)
Supplement: S8 Table — Notes: Results are reported for the calibrated HGB model. All time-dependent features were recomputed within the corresponding observation window. (PDF) [file pone.0352867.s010.pdf]

S8 Table. Window sensitivity with bootstrap 95% confidence intervals.

Window	Accuracy	F1	ROC–AUC	PR–AUC	Brier	ECE15
14d (2w)	0.780 [0.767, 0.791]	0.441 [0.413, 0.470]	0.727 [0.709, 0.743]	0.556 [0.527, 0.586]	0.162 [0.156, 0.169]	0.026 [0.021, 0.040]
28d (4w)	0.814 [0.801, 0.824]	0.570 [0.543, 0.597]	0.794 [0.777, 0.809]	0.664 [0.637, 0.689]	0.141 [0.135, 0.148]	0.036 [0.028, 0.048]
42d (6w)	0.811 [0.800, 0.822]	0.593 [0.570, 0.617]	0.804 [0.789, 0.820]	0.675 [0.649, 0.701]	0.139 [0.133, 0.146]	0.038 [0.033, 0.051]

Notes: Results are reported for the calibrated HGB model. All time-dependent features were recomputed within the corresponding observation window.
